# T43. TRANSCRANIAL DIRECT-CURRENT STIMULATION (TDCS) IN PATIENTS WITH ULTRA-TREATMENT-REFRACTORY AUDITORY HALLUCINATIONS

**DOI:** 10.1093/schbul/sby016.319

**Published:** 2018-04-01

**Authors:** Jean-Pierre Lindenmayer, Tania Sultana, Amandeep Kaur, Rang Yang, Anzalee Khan, Mila Kirstie-Kulsa, Isidora Ljuri, Benedicto Parker

**Affiliations:** 1 New York University; 2 Manhattan Psychiatric Center; 3 Columbia University

## Abstract

**Background:**

Transcranial direct-current stimulation (tDCS), a noninvasive neurostimulation treatment, has been reported to show improvements in treatment-resistant auditory hallucinations in patients with schizophrenia. tDCS administered over a limited number of sessions effectively produced lasting attenuation of auditory hallucinations in otherwise stable outpatients. It has also been shown that tDCS may be a useful intervention for ameliorating cognitive deficits in patients with chronic schizophrenia. The purpose of this study was to test tDCS for auditory hallucinations in ultra-treatment resistant schizophrenia to assess if this form of neurostimulation can alleviate treatment-refractory auditory hallucination symptoms up to 4 weeks after the final treatment. In addition, we also wanted to examine the effects of tDCS on cognitive functions.

**Methods:**

28 inpatients with DSM-V schizophrenia and long-standing treatment resistance and persistent auditory verbal hallucinations were recruited. Each individual participated in behavioral assessments at baseline, endpoint and follow-up [PANSS and Auditory Hallucinations Rating Scale (AHRS) and MCCB cognitive battery] and were randomized to receive active vs. sham tDCS treatments. For active treatment, patients had the inhibitory (cathodal) tDCS electrode placed over left auditory cortex relative to an excitatory (anodal) electrode placed over frontal cortex on the right side. tDCS treatments took place for 20 min twice daily for 5 consecutive days. Assessment batteries were repeated following the 4 weeks of treatment. The Chattanooga, dual channel CHA-1335 stimulator with two 7 × 5 cm (35 cm2) sponge electrodes soaked in a saline solution (0.9% NaCl) was used for the delivery of 2 mA current.

**Results:**

A total of 28 subjects were enrolled (tDCS, n = 13; Control, n = 15). 20 subjects completed the trial. 3 subjects dropped out of the active tDCS treatment group, while 4 subjects did not complete the control treatment due to early discharge from the hospital. Most subjects were male (tDCS n = 10, 76.9%; Control n = 6, 40.0%). Length of present psychiatric admission ranged from 1–25 months, with a mode of 2 months (n = 12) and average of 2.9 months. Participating inpatients were on clozapine, haloperidol, paliperidone depot, fluphenazine decanoate, paliperidone, olanzapine, and risperidone as primary medications. Repeated Measures ANOVA showed a significant difference for the auditory hallucination total score, frequency and number of voices over time (p < 0.05) with greater reduction in scores observed for the tDCS group. Improvements were maintained after 4 weeks. There was no significant change over time observed for the PANSS positive symptoms or total score, or for the PANSS Hallucinatory Behavior item score. When assessing cognitive functioning, only Working Memory change was significant (p = 0.048) between the tDCS and the Control group with the tDCS group showing significant improvement in T-Score as compared to the Control group.

**Discussion:**

Subjects who received tDCS treatment showed a significant reduction in the frequency, number of voices, and total scores of their auditory hallucination. Additionally, subjects in the tDCS group showed significant improvement in the Working Memory. Our results indicate that patients who have been ultra-resistant to antipsychotic treatments and who received tDCS treatment presented with robust diminution of their auditory hallucinations. We conclude that tDCS seems to be effective not only for ambulatory, higher functioning patients, but also for much lower functioning patients with medication-refractory auditory verbal hallucinations.

